# A Note on Exact Differences between Beta Distributions in Genomic (Methylation) Studies

**DOI:** 10.1371/journal.pone.0097349

**Published:** 2014-05-13

**Authors:** Emanuele Raineri, Marc Dabad, Simon Heath

**Affiliations:** Statistical Genomics, Centro Nacional de Análisis Genómico, Barcelona, Catalonia, Spain; Peking University Cancer Hospital and Institute, China

## Abstract

We apply a known algorithm for computing exactly inequalities between Beta distributions to assess whether a given position in a genome is differentially methylated across samples. We discuss the advantages brought by the adoption of this solution with respect to two approximations (Fisher's test and Z score). The same formalism presented here can be applied in a similar way to variant calling.

## Introduction

Average DNA methylation at a locus can be measured by Whole Genome Bisulfite Sequencing (WGBS), which determines the fraction of DNA strands methylated at any given genomic position in a population of cells (this definition is likely to sound obvious to those who already know about WGBS and too terse to those who don't: a good introduction to this kind of measurements is contained in chapter 11 of [1]). In what follows we will call this fraction 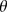; when we distinguish between different samples we will write 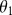 and 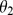. WGBS experiments estimate this numbers by measuring the methylation state of a random ( *i.e.* selected in some unpredictable way) set of reads sequenced from the sample. Since one can only analyze a finite number of reads per sample the value of 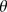 will be known only up to some variability.

In this paper we propose an answer to the basic question : how does one assess whether two cell populations have different methylation levels at a genomic position? Researchers in the field have already dealt with this issue in a variety of ways: for example [2] uses a Fisher's test. In [3] Sun et al. compute a confidence interval for 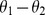 starting from some reasonable choice of a probabilistic model. Bsmooth [4] (which tackles the slightly different problem of defining differently methylated *regions* as opposed to positions) ultimately relies on a t-test. The authors of [5] use a hierarchical model to estimate the parameters needed for a Gaussian hypothesis test. Here we would like to bring attention to another possible approach, based on properties of the Beta distributions which are explained in [6], [7]. Similarly to *e.g.* [3] we do not test an hypothesis and output a p-value; rather we compute the probability distribution of the parameter of a Bayesian model.

### Beta Distribution to Model Methylation Probabilities

The Beta probability distribution (over 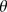) with parameters 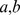 is defined by
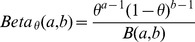
where 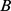 is the Beta function



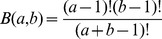


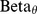
 appears very naturally in many studies of genomic data: typically such analyses also entail the comparison between different samples, which in turn means that different Betas have to be combined. Here for concreteness we are describing the case of measuring DNA methylation differences across samples via whole genome bisulfite sequencing but the same concepts apply with almost no change to genotyping.

To appreciate how this variability can be quantified, consider a set of reads out of a WGBS experiment covering a certain genomic coordinate 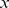 with read depth 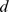. Since not all the strands in the sample being sequenced will, in general, have the same bases methylated at the same time, this will be a collection of heterogeneous reads : some will indicate methylation at position 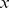 (these are the so called *non converted* reads), others (the *converted* reads) will correspond to molecules that are not methylated. Now, if 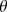 were known a priori, the probability of obtaining 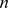 non converted reads would be given by a binomial distribution (which is closely related to 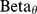):
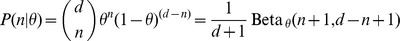



If one assumes a uniform prior on 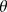, (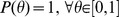) the expression for 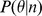 is very similar (The factor 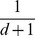 cancels out when applying Bayes' theorem)
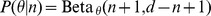



Therefore, to assess whether a position is differentially methylated across two samples with non converted reads respectively 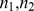 and read depths 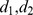 one has to compute
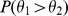
where



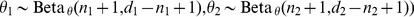
(1)The purpose of the software we will discuss in this note is to estimate 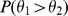 given the result of a WGBS experiment.

### Exact Computation of Beta Differences

A method for computing
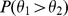
which turns out to be efficient enough for our purposes is presented in full detail in [6], [7]. We will summarize its derivation here for the sake of completeness, and advise interested readers to study those papers for a more detailed discussion. We start with some preliminary definitions: let 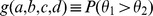 where 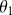 and 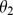 are distributed respectively as 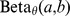 and 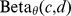. Besides, we will use the notation 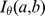 for the cumulative distribution function of the Beta distribution (also known as the incomplete Beta distribution).

Now, by definition one has
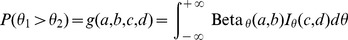



But then, using the identity ([8])
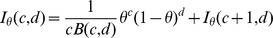
one finds that
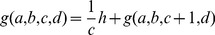
(2)where



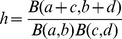



Furthermore, one can prove that 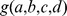 possesses a number of symmetries. An obvious one is 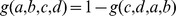. Also true are
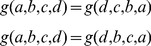
(3)


Using (2) and (3) one can design a nice recursive scheme
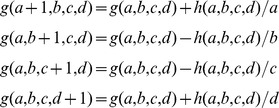
where the base case is provided by 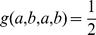 (this because if 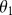 and 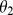 have exactly the same distribution, 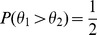).

### Approximate Computation

Even if methylation data are well modelled by a 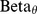, the comparison presented above is never (to our knowledge) used in the literature. As (hopefully fair) representatives of the methods which we have found are used instead, we will analyze the performances of the Fisher's test and that of a test based on a Gaussian approximation.

To do a Fisher's test, one builds a contingency table with the number of non converted and converted reads in the two samples (note that this kind of test breaks down when one of the rows (or columns) of the contingency table is zero). In the Gaussian approximation, one models 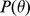 for each sample with a Gaussian with the same mean and variance of 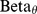; and then uses the two Gaussians to test for differences between 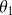 and 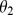. In both cases we will consider one tailed tests.

## Results and Discussion

### Comparison with Approximate Results

We organized the comparison between the exact and approximate solution in two steps. First, we looked at the behaviour of the two tests on a pair of real samples (see below for instructions on how to access the data we used).

**Figure 1:**
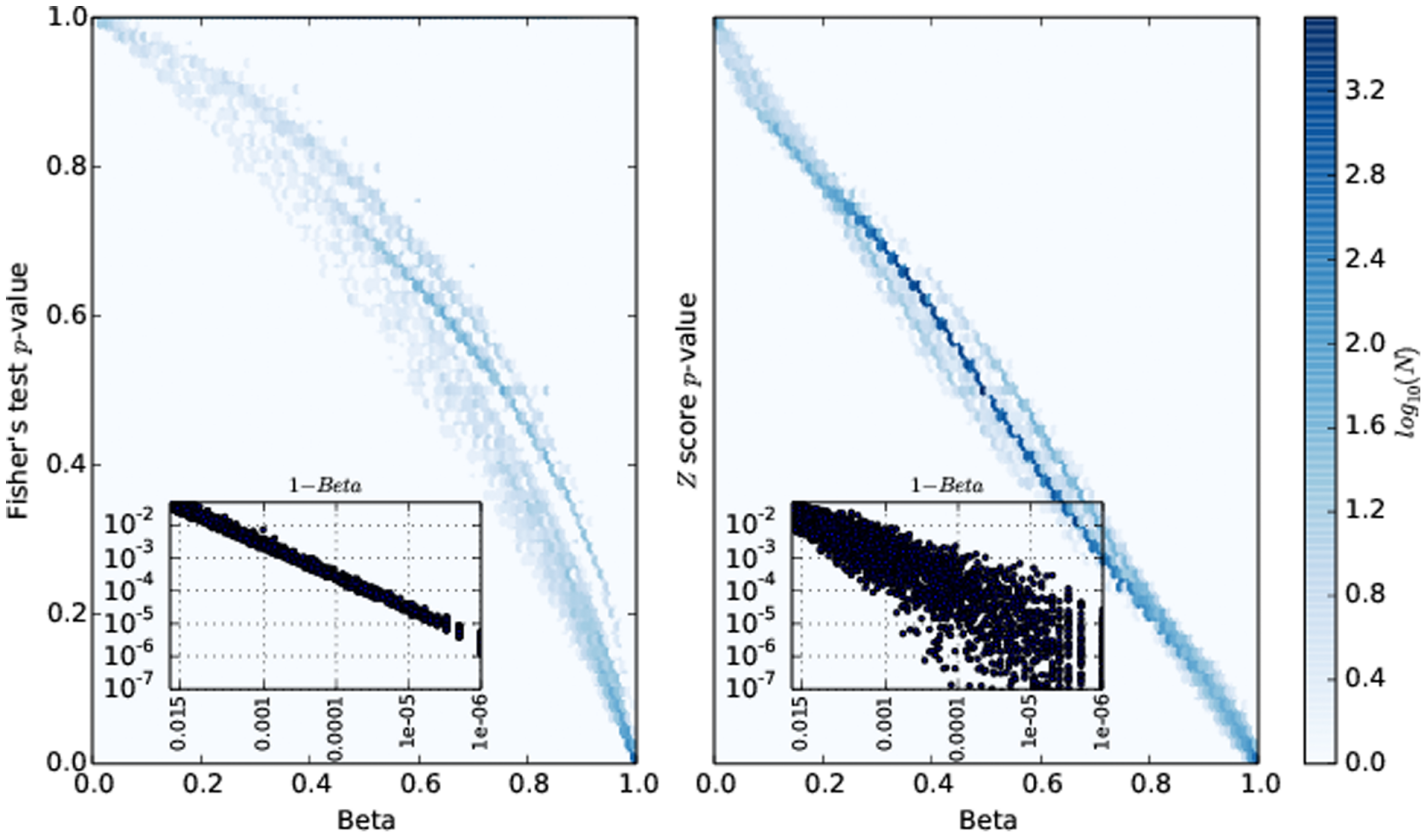
Comparing beta distribution with Fisher's test and Z score test. Each plot contains an enlarged version around p-value 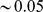. Notice that the in these magnified plots the 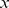 axis is 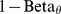, for exact powers of 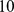 take less space in the labels then string of 9 s.

The results are shown in figure 1. On the 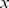 axis we plotted 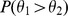, on the 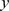 axis we plotted the corresponding 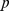-value obtained by approximating the Beta respectively with a Fisher's test (on the left) and with a Gaussian (on the right). We did the comparison over 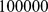 positions : the plot is in fact a two dimensional histogram, in which different shades of blue indicate how many times the two values fall into a certain region of the plane. There is not much to comment there except to note that, as expected, there is a broad correspondence between the different methods. Also, at such a scale the Beta probabilities seem more similar to the Z score test than to the Fisher's p-values (the right hand side plot looks more like a diagonal).

**Figure 2:**
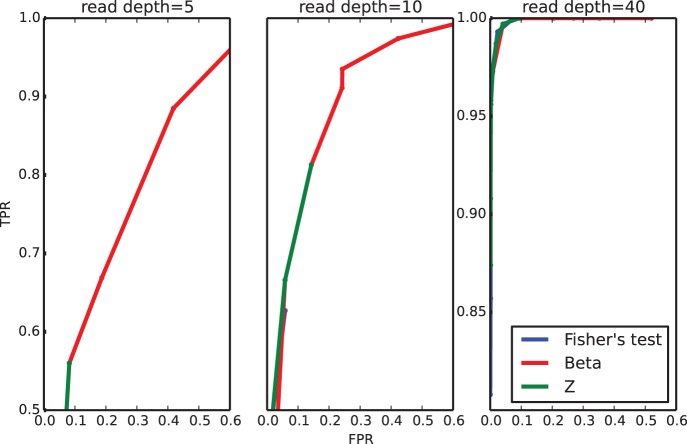
ROC curves for the three methods under comparison. Each point in the ROC curve is obtained by choosing a different threshold for calling differential methylation. For the Z score test and the Fisher's test the p-values are: 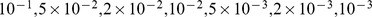. For the Beta distributions the threshold probabilities are: 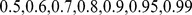. TPR means true positive rate; FPR means false positive rate.

**Figure 3:**
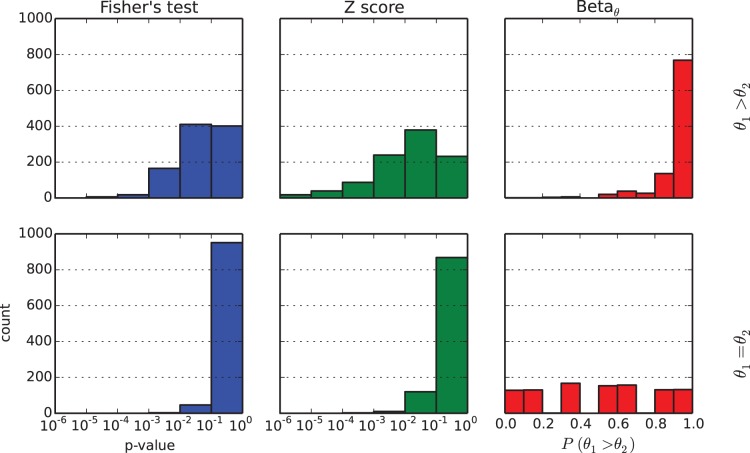
Distribution of p-values (for the hypothesis tests discussed) and of 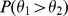 computed with the 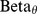 model. The first row depicts the truly different samples (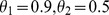). The bottom row refers to the control samples. For all the plots 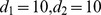.

Next, we simulated a pair of samples whose counts are generated by the same underlying binomial process (*i.e.* 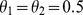) at different coverages. These constitute a negative control, in the sense that none of the methods should report a significant difference between the samples. Furthermore, we generated a pair of samples such that their underlying binomial probabilities are markedly different 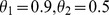; those are the true positives, *i.e.* cases for which the tests should detect that 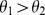. We then compare the receiver operating characteristic (ROC) curves of the three methods for different values of the samples' coverages, 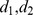. The results are depicted in figure 2. That plot justifies the usage of the 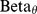 distribution: the number of false negatives accumulated by the other two methods considered stops them from reaching an high enough true positive rate (even when the threshold for computing it is very permissive). Note, for example, that the blue line is not even visible in the leftmost panels of figure 2. This effect is also shown in figure 3 where we depict the distribution of the outputs for the three methods at read depth 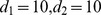.

### Differentially Methylated Regions and Effects of Coverage

Using the above concepts, we can compute differentially methylated regions (DMR) along the genome : these are uninterrupted blocks of nucleotides where the two samples have different methylation. One possible technique to find such blocks is to conjoin a number of adjacent nucleotides in a DMR, disregarding their exact methylation probabilities, and to assign hard boundaries. This usually implies that a number of *ad hoc* rules must be established to control the minimum distance between 2 neighbouring DMRs, the minimum length of a DMR, how to exactly count the intersection of DMRs with annotated regions, and so on and so forth. Using our method, though, one can simply assign to each nucleotide the probability computed by the algorithm presented here; any further analysis can be conducted without imposing arbitrary threshold or boundaries. For instance one can ask what is the average value of this probability over some specific regions (introns, enhancers) with respect to randomly chosen regions of the genome. Often it is not clear a priori what is the correct scale to use when looking at methylation : if this is the case, one can smooth the probability per nucleotide by computing a kernel density estimation at various bandwidths, or simply clump together the values of a number of nearby bases in a single (average) value. Note that smoothing is justified by the fact that methylation levels are correlated in space (the strength and persistence of the correlation is different from sample to sample, reflecting technical and biological variability); in fact as hinted at in [4], analyzing together nearby positions could provide a way of correcting measurement errors.

**Figure 4:**
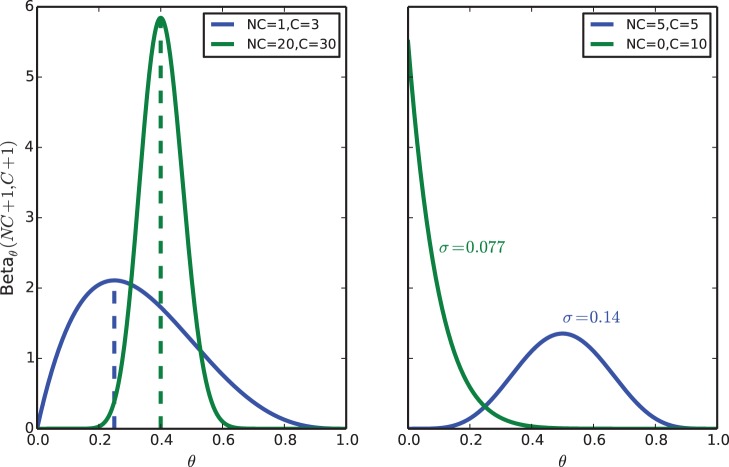
Effects of coverage on 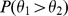. In the left panel we show that a sample for which the methylation is estimated (with high uncertainty) to be low can be (with some probability) more methylated than a sample for which the methylation level is higher, and certain. In the right panel : even if the total coverage is the same, the uncertainty over 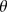 varies according to the count of non converted (NC) and converted (C) reads.

We would also like to comment on the fact that the different coverage of the samples does have an effect on the estimation of differential methylation. The main idea to understand here is that low coverage means uncertainty: and uncertainty can give rise to results which, while correct, are slightly counterintuitive. For example in figure 4 we show that a sample with low methylation and low coverage can be (maybe, one cannot say for sure) more methylated than a sample with high, certain methylation. The right panel of the same figure suggests that a good way of filtering for certainty is to select positions with low estimated variability (rather than to select based on read counts): this is because the same read depth can correspond to different variances depending on how many reads are non converted or converted.

Finally, once one has the estimates for 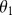 and 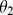 (as obtained via the ratio of unconverted reads over the coverage) and 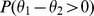 ( *i.e.* the output of the algorithm expalined in this paper) one can take an informed decision on a locus, keeping into account both the size of the difference in methylation and its variability.

### Implementation and Data Availability

The algorithm described above is implemented in a C program, called methyl_diff, available from the Github page of one of the authors : http://emanueleraineri.github.io/. The program takes as input (from stdin) four integers, *i.e.* the number of non converted and converted reads for the first and the second sample respectively, and prints 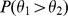 on the stdout. It takes 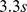 to process 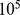 lines on off-the-shelf hardware (MacBookPro with Intel i7@2.66 GHz). Note that the data used to produce figure 1 are publicly available (they were generated for BLUEPRINT, a consortium, studying epigenetic marks in immune system cells.) in at least two ways (also corresponding to two different formats):

1. First of all, they can be downloaded from the same web page where the source code of our implementation is stored. The file G199.G202 contains the methylation levels of 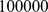 random positions from the chromosome 1 of samples G199 and G202 (first we determined which positions had been sequenced in both samples; then we extracted a random subset of those). One can feed columns 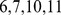 directly to the methyl_diff executable (those columns are the unconverted, converted reads from the two samples).
2. Secondly, they can be downloaded from the BLUEPRINT project ftp site ftp.ebi.ac.uk/pub/databases/blueprint/data/homo_sapiens/.
